# Effect of Intracameral Air Bubble Injection on Early Visual and Intraocular Pressure Outcomes After Phacoemulsification

**DOI:** 10.7759/cureus.101800

**Published:** 2026-01-18

**Authors:** Gede Pardianto, Stefenie Stefenie, Givenchy A Winarjo, Fajri Ijrian, Diyah Purworini

**Affiliations:** 1 Ophthalmology and Vision Sciences, Universitas Prima Indonesia, Medan, IDN; 2 Biomedical Sciences, Universitas Prima Indonesia, Medan, IDN

**Keywords:** cataract surgery, corneal edema, intracameral air bubble injection, phacoemulsification, visual acuity

## Abstract

Purpose: To evaluate the efficacy of intracameral air bubble injection in enhancing visual acuity following phacoemulsification cataract surgery.

Methods: A prospective quasi-experimental study was conducted on 98 eyes from 78 participants who underwent phacoemulsification at SMEC Eye Hospital, Medan, between March and August 2025. All surgeries were performed by a single surgeon using the phaco-chop technique with bimanual irrigation/aspiration and intraocular lens (IOL) hydro-implantation using a Venturi phaco machine (Bausch+Lomb, Bridgewater, NJ, USA). At the conclusion of the surgery, 0.5% levofloxacin was administered intracamerally for infection prophylaxis, followed by air bubble injection. Postoperative outcomes included uncorrected visual acuity (UCVA), best-corrected visual acuity (BCVA), and intraocular pressure (IOP), analyzed using paired t-tests (95% CI; p<0.05).

Results: UCVA improved significantly from logMAR 0.823 ± 0.113 (0.602-1.301) to 0.103 ± 0.073 (0.000-0.397) (p<0.001). BCVA improved from logMAR 0.651 ± 0.108 (0.477-0.903) to 0.083 ± 0.079 (0.000-0.176) (p<0.001). Mean IOP decreased from 17.23 ± 1.52 mmHg (16-20) to 13.26 ± 1.21 mmHg (11-16) (p<0.001). Minimal corneal edema and no significant inflammation were observed.

Conclusion: Intracameral air bubble injection at the end of phacoemulsification significantly improves UCVA and BCVA, lowers IOP, and minimizes corneal edema. This simple, safe, and reproducible technique represents a valuable adjunct to cataract surgery for optimizing postoperative visual outcomes and patient satisfaction.

## Introduction

Cataract surgery remains one of the most common and effective surgical procedures performed globally, particularly phacoemulsification, which employs ultrasound energy to emulsify and remove the opacified lens. Postoperative outcomes are generally favorable [1]; however, complications such as corneal edema can significantly affect visual recovery [1,2]. Corneal edema results primarily from endothelial cell loss, often exacerbated by intraoperative surgical trauma during phacoemulsification [2,3].

The introduction of techniques such as intracameral air bubble injection has emerged as a potential approach to enhance corneal clarity and subsequently improve visual acuity after surgery [3]. Despite advances in surgical methods, the specific role of air bubble injection in minimizing corneal edema and optimizing postoperative visual outcomes remains insufficiently explored [2,3]. Maintaining corneal transparency following surgery is critical, as it directly correlates with patient satisfaction and quality of life [4].

This study aims to evaluate the effect of intracameral air bubble injection on early visual acuity, intraocular pressure (IOP), and corneal clarity following phacoemulsification.

## Materials and methods

Study design and setting

A single-arm prospective interventional study was conducted at SMEC Eye Hospital, Medan, from March to August 2025, and included 98 eyes from 78 patients undergoing phacoemulsification. The study adhered to the tenets of the Declaration of Helsinki and received approval from the SMEC Hospital Research Ethical Review Committee (approval number: EA00000688, February 21, 2025). Written informed consent was obtained from all participants.

Sample size calculation

The sample size was calculated based on the expected change in best-corrected visual acuity (BCVA, logMAR), which was defined as the primary outcome of the study. A paired t-test was used with a significance level of α = 0.05 and a statistical power of 80%. A minimal clinically important difference (MCID) of 0.1 logMAR was assumed, consistent with commonly accepted clinical thresholds for meaningful visual improvement, along with variability reported in previous studies. Based on these assumptions, the minimum required sample size was approximately 90 eyes. To account for an estimated 10% potential loss to follow-up, a total of 98 eyes were included in the study.

Participants

Inclusion criteria required patients to have physiological cataract (excluding traumatic, complicated, and pseudoexfoliation cataracts), BCVA worse than 20/70, anatomically normal ocular structures (conjunctiva, cornea, and retina), and IOP below 21 mmHg. Patients with systemic disease or ocular abnormalities were excluded.

Exclusion criteria such as marked patient anxiety and prolonged surgical duration (>15 minutes) were defined a priori to standardize surgical conditions and minimize intraoperative variability.

Surgical technique: phacoemulsification with intracameral air bubble injection

Preoperative Preparation

Optical biometry was performed using the IOLMaster 500 (Carl Zeiss Meditec, Dublin, CA, USA) for intraocular lens (IOL) power calculation. Pupillary dilation was achieved with 1% tropicamide (Mydriatil; PT Cendo, Bandung, Indonesia), and topical anesthesia was administered with 0.5% tetracaine hydrochloride (Pantocain; PT Cendo). Sterility protocols included immersion of extraocular instruments in 5% povidone-iodine, eyelid and skin disinfection with 10% povidone-iodine, and conjunctival disinfection with 5% povidone-iodine.

Phacoemulsification

All surgeries were performed by a single surgeon (GP) using the Stellaris Elite Venturi phaco machine (Bausch+Lomb, Bridgewater, NJ, USA). The phaco-chop technique was employed, followed by bimanual irrigation/aspiration and IOL hydro-implantation.

Intracameral Injection

At the end of surgery, 0.5% levofloxacin (Optiflox; PT Erela, Semarang, Indonesia) was injected intracamerally for infection prophylaxis. This was followed by intracameral air bubble injection. Surgical time ranged from four to eight minutes.

At the completion of phacoemulsification and IOL implantation, a small volume of sterile air (approximately 0.1-0.15 mL) was injected into the anterior chamber using a cannula. The volume was intentionally limited to achieve partial anterior chamber fill, sufficient to cover the central corneal endothelium and visual axis while avoiding contact with the iris.

This approach was chosen to minimize the risk of pupillary block or iris bombe. In the supine postoperative position, the air bubble was expected to rise anteriorly, allowing coverage of the corneal endothelium without exerting posterior pressure on the iris. Complete anterior chamber fill was avoided to maintain aqueous circulation and anterior chamber stability.

In routine postoperative follow-up, the intracameral air bubble was observed to resolve spontaneously within approximately three days.

Postoperative Care

Patients were instructed to avoid exposure to water, dust, smoke, wind, and other potential ocular irritants. Protective glasses were provided for daytime use, and an eye shield was recommended during sleep. Postoperative medications consisted of topical 0.5% levofloxacin (LFX; PT Cendo) and 0.1% sodium diclofenac (Noncort; PT Cendo), each administered six times daily. No systemic medications were prescribed. All patients followed the same postoperative care protocol and were examined on postoperative day one and day seven.

Postoperative anti-inflammatory management relied exclusively on topical diclofenac, in accordance with local clinical practice in tropical settings, where cautious use of topical corticosteroids is commonly adopted because of concerns regarding postoperative infection and cystoid macular edema. Nevertheless, the absence of corticosteroids may have influenced postoperative inflammation and corneal clarity and therefore represents a potential confounding factor. This limitation should be considered when interpreting the findings, and future comparative studies incorporating alternative anti-inflammatory regimens, including corticosteroids, may help further clarify their impact on corneal outcomes.

Outcome measures

Outcome measures included uncorrected visual acuity (UCVA), BCVA (logMAR), and IOP (mmHg), assessed preoperatively and at one week postoperatively. Data were analyzed using paired t-tests, with statistical significance set at p < 0.05 (95% CI). Analyses were performed using SPSS version 29.0.2.0 (IBM Corp., Armonk, NY, USA).

## Results

A total of 98 eyes from 78 participants underwent phacoemulsification following meticulous patient selection. The study population had a mean age of 65.7 years (standard deviation 14.6 years), comprising 35 (44.87%) male and 43 (55.13%) female participants. Laterality analysis showed that 48 eyes (48.98%) were right eyes and 50 eyes (51.02%) were left eyes. Ethnic distribution revealed Malay participants accounted for 39 eyes (50%), Chinese for 32 eyes (41.03%), and Indian for seven eyes (8.97%) (Table 1).

**Table 1:** Baseline characteristics Sex, laterality, and race are presented as number (percentage).

Characteristic	Value
Sex	n (%)
Male	35 (44.87%)
Female	43 (55.13%)
Laterality	n (%)
Right eye	48 (48.98%)
Left eye	50 (51.02%)
Race/Ethnicity	n (%)
Malay Indonesian	39 (50.00%)
Chinese Indonesian	32 (41.03%)
Indian Indonesian	7 (8.97%)

Significant improvements were observed in both UCVA and BCVA postoperatively (p < 0.001 for both). Mean UCVA improved from logMAR 0.823 ± 0.113 (0.602-1.301) preoperatively to logMAR 0.103 ± 0.073 (0.000-0.397) postoperatively, while BCVA improved from logMAR 0.651 ± 0.108 (0.477-0.903) to logMAR 0.083 ± 0.079 (0.000-0.176). Clinically minimal corneal edema, well-formed anterior chambers, and absence of significant postoperative inflammation were observed during follow-up. IOP also decreased significantly (p < 0.001), from a preoperative mean of 17.23 ± 1.52 mmHg (16-20) to 13.26 ± 1.21 mmHg (11-16) postoperatively (Table 2).

**Table 2:** Comparison of visual acuity and intraocular pressure (IOP) following phacoemulsification with air bubble injection Values are presented as mean ± standard deviation with range (minimum–maximum). All comparisons were performed using the paired t-test. UCVA: uncorrected visual acuity, BCVA: best-corrected visual acuity

Variable	Preoperative (Mean±SD, Range)	One-week postoperative (Mean±SD, Range)	p
UCVA (logMAR)	0.823±0.113 (0.602-1.301)	0.103±0.073 (0.000-0.397)	<0.001
BCVA (logMAR)	0.651±0.108 (0.477-0.903)	0.083±0.079 (0.000-0.176)	<0.001
IOP (mmHg)	17.23 ± 1.52 (16-20)	13.26 ± 1.21 (11-16)	<0.001

**Figure 1:**
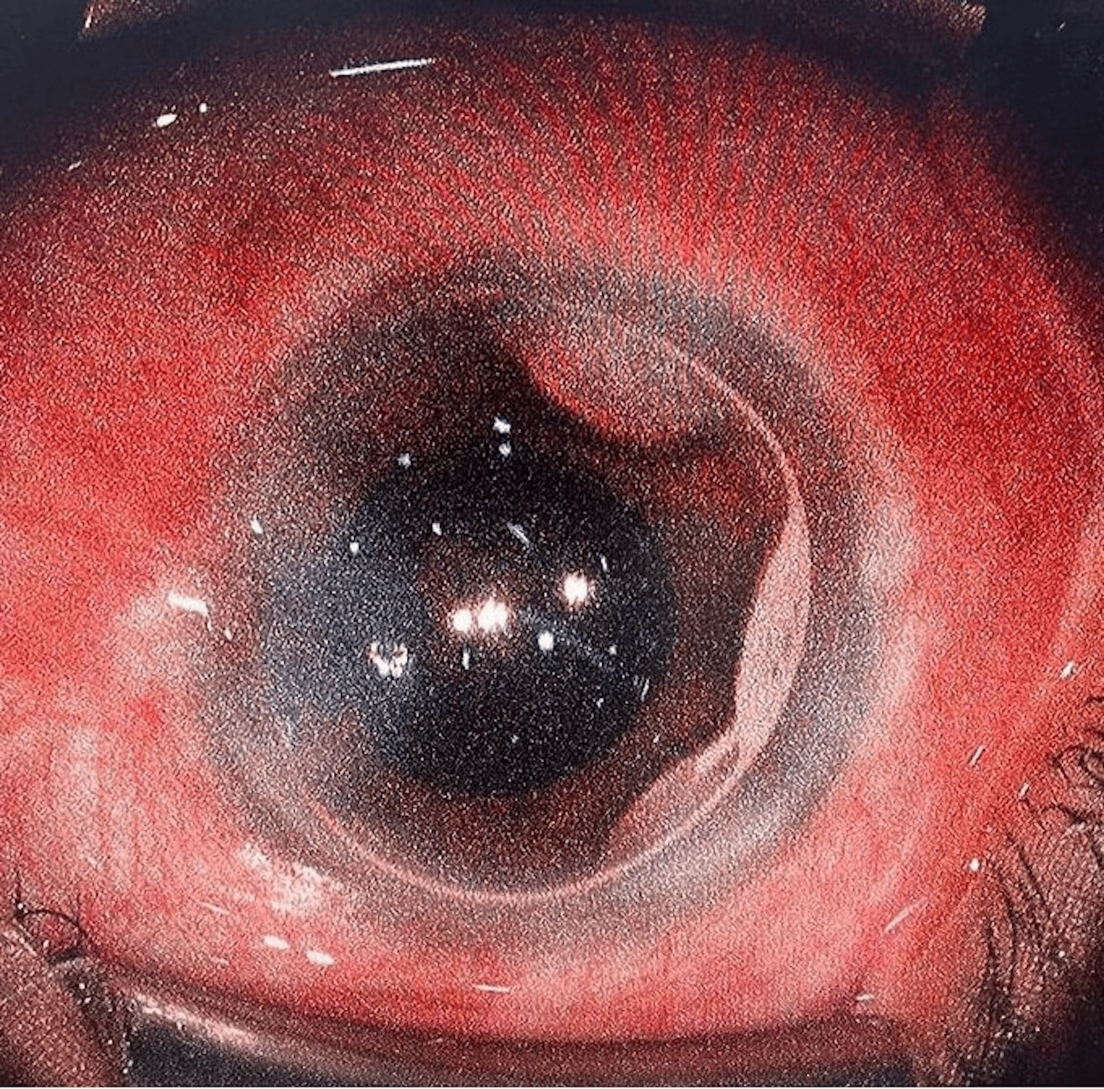
Intracameral air bubble at the end of phacoemulsification. A small air bubble is injected into the anterior chamber at the end of surgery to help maintain chamber stability and promote postoperative corneal clarity.

These findings demonstrate favorable early postoperative visual acuity outcomes, stable anterior chamber configuration, and reduced IOP in this cohort following phacoemulsification with adjunct intracameral air bubble injection (Figure 1).

## Discussion

The findings of this study align with previous research indicating that corneal edema is a significant factor affecting visual outcomes following cataract surgery [1,2,4]. Intracameral air bubble injection has been described in prior studies as an adjunct technique associated with favorable early postoperative corneal clarity and visual outcomes [2-4]. Proposed mechanisms in the literature include temporary coverage of the corneal endothelium and postoperative corneal clarity; however, these mechanisms were not directly evaluated in the present study [3,4].

In the present study, significant improvements were observed in both UCVA and BCVA. Mean UCVA improved from logMAR 0.823 ± 0.113 (0.602-1.301) to logMAR 0.103 ± 0.073 (0.000-0.397), while BCVA improved from logMAR 0.651 ± 0.108 (0.477-0.903) to logMAR 0.083 ± 0.079 (0.000-0.176), as shown in Table 2. These improvements are consistent with the expected outcomes of uncomplicated phacoemulsification and IOL implantation [1,5-8]. Clinically minimal corneal edema was observed during early follow-up and did not interfere with visual recovery, allowing for significant improvement in visual acuity within one week. Corneal clarity at one week postoperatively was observed in conjunction with intracameral air bubble use at the conclusion of surgery [2-4,9,10]. Given the absence of a comparison group, these findings should be interpreted as observational.

A significant reduction in IOP was also observed (p < 0.001), with mean preoperative values decreasing from 17.23 ± 1.52 mmHg (16-20) to 13.26 ± 1.21 mmHg (11-16) postoperatively (Table 2). This reduction is consistent with known physiological changes following cataract extraction. While previous studies have suggested that intracameral air may be associated with anterior chamber configuration changes and aqueous humor dynamics, the present study design does not allow attribution of IOP changes specifically to air bubble injection. Given the bidirectional relationship between elevated IOP and corneal edema [3,11], the observed IOP reduction may be relevant when interpreting early postoperative visual outcomes in this cohort [2,11].

Intracameral administration of antibiotics at the end of surgery has been shown to reduce postoperative infection risk and inflammation [12,13]. In this study, postoperative inflammation appeared clinically minimal during early follow-up, which may have supported early visual recovery in the setting of adequate corneal clarity [13,14].

The standardized nature of the surgical technique and postoperative management across all cases supports consistency of outcome assessment. However, several limitations should be acknowledged. The absence of a control group undergoing phacoemulsification without intracameral air bubble injection limits causal inference, as improvements in visual acuity and IOP are expected following uncomplicated cataract surgery. In addition, corneal edema was assessed clinically and was not included as a quantitative outcome variable; objective measurements such as pachymetry or standardized corneal edema grading were not performed, limiting objective evaluation of corneal clarity.

Furthermore, postoperative anti-inflammatory management relied exclusively on topical diclofenac without corticosteroids. While this regimen reflects local clinical practice, it may have influenced postoperative inflammation and corneal clarity and therefore represents a potential confounding factor. The relatively small sample size and short follow-up period further limit long-term interpretation. Future comparative studies with larger cohorts, longer follow-up, objective corneal outcome measures, and alternative postoperative anti-inflammatory regimens, including corticosteroids, are warranted to further clarify the role of intracameral air bubble injection in the early postoperative period following phacoemulsification.

## Conclusions

Intracameral air bubble injection at the conclusion of phacoemulsification was associated with favorable early postoperative outcomes, including good visual acuity recovery, clinically minimal corneal edema, and lower IOP. When applied as part of a standardized surgical and postoperative protocol, this simple and low-cost adjunct may support early visual rehabilitation following uncomplicated cataract surgery. The technique is easy to perform and may represent a practical addition to routine phacoemulsification in everyday clinical practice.
